# Understanding social media discourse on antidepressants: unsupervised and sentiment analysis using X

**DOI:** 10.1192/j.eurpsy.2025.10

**Published:** 2025-03-05

**Authors:** Juan Pablo Chart-Pascual, Javier Goena, Francisco Lara, María Montero Torres, Julen Marin Napal, Rodrigo Muñoz, Cielo García Montero, Oscar Fraile Martínez, Miguel Ángel Ortega, Gonzalo Salazar de Pablo, Ana González Pinto, Javier Quintero, Melchor Alvarez-Mon, Miguel Ángel Álvarez-Mon

**Affiliations:** 1 Psychiatry Department, Osakidetza Basque Health Service, Araba University Hospital, Vitoria-Gasteiz, Spain; 2 Bioaraba Research Institute, Vitoria-Gasteiz, Spain; 3 University of the Basque Country UPV/EHU, Vitoria-Gasteiz, Spain; 4 Centro de Investigación en Red de Salud Mental (CIBERSAM), Madrid, Spain; 5 Psychiatry Department, Basurto University Hospital, Osakidetza Basque Health Service, Bilbao, Spain; 6 Biobizkaia Health Research Institute, OSI Bilbao-Basurto, Bilbao, Spain; 7 Department of Signal Theory and Communications and Telematic Systems and Computing, School of Telecommunications Engineering, Rey Juan Carlos University, Madrid, Spain; 8 Department of Medicine and Medical Specialties, University of Alcala, Alcalá de Henares, Spain; 9 Department of Psychiatry and Mental Health, Hospital Universitario Infanta Leonor, Madrid Spain; 10 Ramón y Cajal Institute of Sanitary Research (IRYCIS), Madrid, Spain; 11 Immune System Diseases-Rheumatology and Internal Medicine Service, Centro de Investigación Biomédica en Red Enfermedades Hepaticas y Digestivas, University Hospital Príncipe de Asturias, Alcala de Henares, Spain; 12 Department of Child and Adolescent Psychiatry, Institute of Psychiatry, Psychology & Neuroscience, King’s College London, London, UK; 13 Child and Adolescent Mental Health Services, South London and Maudsley NHS Foundation Trust, London, UK; 14 Department of Child and Adolescent Psychiatry, Institute of Psychiatry and Mental Health. Hospital General Universitario Gregorio Marañón School of Medicine, Universidad Complutense, IiSGM, CIBERSAM, Madrid, Spain; 15 Department of Legal and Psychiatry, Complutense University, Madrid, Spain

**Keywords:** antidepressants, esketamine, selective serotonin reuptake inhibitors, sentiment analysis, twitter (X)

## Abstract

**Background:**

Antidepressants are essential in managing depression, including treatment-resistant cases. Public perceptions of these medications, shaped by social media platforms like X (formerly Twitter), can influence treatment adherence and outcomes. This study explores public attitudes toward antidepressants through sentiment and topic modeling analysis of tweets in English and Spanish from 2007 to 2022.

**Methods:**

Tweets mentioning antidepressants approved for depression were collected. The analysis focused on selective serotonin reuptake inhibitors (SSRIs) and glutamatergic drugs. Sentiment analysis and topic modeling were conducted to identify trends, concerns, and emotions in discussions across both languages.

**Results:**

A total of 1,448,674 tweets were analyzed (1,013,128 in English and 435,546 in Spanish). SSRIs were the most mentioned antidepressants (27.9% in English, 58.91% in Spanish). Pricing and availability were key concerns in English tweets, while Spanish tweets highlighted availability, efficacy, and sexual side effects. Glutamatergic drugs, especially esketamine, gained attention (15.61% in English, 25.23% in Spanish), evoking emotions such as fear, sadness, and anger. Temporal analysis showed significant increases in discussions, with peaks in 2012 and 2021 for SSRIs in Spanish, and exponential growth from 2018 to 2021 for glutamatergic drugs. Emotional tones varied across languages, reflecting cultural differences.

**Conclusions:**

Social media platforms like X provide valuable insights into public perceptions of antidepressants, highlighting cultural variations in attitudes. Understanding these perceptions can help clinicians address concerns and misconceptions, fostering informed treatment decisions. The limitations of social media data call for careful interpretation, emphasizing the need for continued research to improve pharmacovigilance and public health strategies.

## Introduction

Depression is a highly prevalent disorder, being a leading cause of mortality, disability, and reduced quality of life worldwide [1–4]. Depressive symptoms vary widely, and they can be expressed in different intensities and forms. Some of depression’s core symptoms include sadness, anhedonia, guilt, low self-esteem, sleep and eating disturbances, fatigue, impaired concentration, suicidal ideation, and suicide attempts [5, 6]. The age of onset is around the mid-20s, but symptoms may be expressed earlier during childhood and adolescence [7]. By 2030, depressive disorders are expected to be the largest cause of disease burden [8].

Antidepressants are a heterogeneous group of drugs primarily indicated for the treatment of depressive disorders, having demonstrated their effectiveness in this area [9–12]. Additionally, these drugs are used in other disorders, such as anxiety, and even in other non-psychiatric conditions, like the treatment of pain or urological diseases [13–15]. In recent years, the global consumption of antidepressants has increased significantly, being the fastest-growing group of psychotropic drugs, with an average annual growth of 3.5% worldwide [9, 10, 16–18]. Selective serotonin reuptake inhibitors (SSRIs) are among the most used pharmacological treatments and are the first-line therapy for most depressive and anxiety disorders. By 2019, the use of SSRIs had doubled that of all other antidepressants combined, especially in developed countries like the United States and across Europe [1]. Alongside SSRIs, other antidepressants, such as serotonin-norepinephrine reuptake inhibitors (SNRIs), tricyclic antidepressants (TCA), and glutamatergic modulators, remain alternative therapeutic options [15]. However, recent controversies have considered that the improvements seen in clinical trials may not fully justify their widespread use [19–21]. Concerns about overprescription, long-term side effects such as sexual dysfunction [22, 23], have garnered increasing attention.

These limitations – such as limited efficacy, significant side effects, and concerns over overprescription – become even more critical in cases of treatment-resistant depression (TRD), a severe form of depression defined as a failure to respond to two or more antidepressant regimens despite adequate dose, duration, and treatment adherence [24]. Some authors advocate for the alternative term “difficult-to-treat depression,” as TRD underscores the inherent limitations of traditional antidepressants in terms of efficacy [25]. TRD affects approximately 28% of people who suffer from depression, leading to an even higher significant loss of quality of life and functionality [25]. However, the literature claims that there are still many difficulties to address regarding the definition and consequences of TRD, representing an important barrier in mechanistic and translational research [26]. Incorporating TRD as a central topic in the broader discussion of depression treatment is essential to reflect clinical realities and to adapt therapies to the individual characteristics of patients.

In this complex landscape, intranasal administration of the glutamatergic modulator esketamine has arisen as a promising agent in the medical management of TRD [15]. However, these new treatments come with economic implications and challenges in terms of access, as the high costs and the need for specialized administration limit their availability to broader populations. The rise of precision medicine and personalized therapies [27] offers a shift toward more tailored approaches to TRD, but addressing disparities in access and affordability is crucial to ensure these advancements reach a broader population.

Despite the widespread use of antidepressants, negative opinions are still common, leading many patients to hesitate before starting treatment, or becoming non-adherent to the prescription during the treatment period. Although 66% of patients had positive views of antidepressants, they are worried about the doses and safety [6]. This reluctance is often driven by negative beliefs and attitudes toward these medications [28, 29]. Traditional methods for studying patient experiences, such as surveys and interviews [30–32], may not capture true sentiments due to desirability bias, where patients tend to present themselves more favorably [33]. As a result, patients may avoid sharing negative views about medications in clinical settings, preferring informal spaces like social media to express their true concerns [34–36].

Social media offers significant benefits, including access to real-time data, a greater diversity of opinions, and the ability to capture a more accurate picture of social perception [37–39]. Research has demonstrated its effectiveness in identifying adverse events and continuous monitoring of medication use [40–44]. X, as a pharmacovigilance tool for detecting adverse events, has gained relevance in recent years, especially in mental health, where its use as a research vehicle is increasing [45, 46]. Previous studies have analyzed changes in X use, identifying increased posting frequency or altered patterns during depressive episodes [47, 48], reinforcing the suitability of social media for studying depression-related behaviors. Studies on the use of psychotropic drugs, psychological therapies, and electroconvulsive therapy have found that descriptions of X, as well as the associated social perceptions and emotions, closely resemble scientific evidence, allowing us a better understanding of public perceptions of different therapeutic approaches in mental health [40, 49, 50]. However, social media algorithms tend to prioritize content with high engagement, which can amplify negative or extreme views, especially about antidepressants, potentially distorting public perception and complicating efforts to promote balanced, evidence-based information.

Although some initial studies have evaluated different perspectives and points related to antidepressants on X [49, 51], the broader social perspectives, especially cross-cultural and multilingual insights, remain understudied. Social and cultural attitudes play a critical role in shaping public perception, adherence, and overall effectiveness of antidepressant treatments, yet there is a notable gap in research examining these factors across diverse linguistic and cultural contexts. This study uniquely tries to address this gap by applying advanced artificial intelligence techniques to analyze public discussions on X in both English and Spanish. By doing so, we aim to shed light on the social and cultural factors that influence the effectiveness of pharmacological treatment for depression.

## Methods

### X data collection search strategy

This study focuses on analyzing tweets related to antidepressants approved for the treatment of depression and TRD. We employed the *Twitter Binder* search engine to collect all public tweets referencing antidepressant medications approved by the Food and Drug Administration (FDA) or the Spanish Agency of Medicines and Medical Devices (AEM) from January 1, 2007, to December 31, 2022. The search covered tweets in both English and Spanish.

The search strategy included both generic drug names and brand names. A full list of the keywords used can be found in the Supplementary Material. The inclusion criteria for the tweets were: (a) They must be public, (b) They must contain at least one of the listed keywords, (c) They must be written in either English or Spanish, and (d) Published between 2007 and 2022.

Due to the relatively limited volume of Spanish tweets mentioning dual agents, other antidepressants, and tricyclics, the analysis was primarily concentrated on the two groups with the highest tweet volumes across both languages: SSRIs and glutamatergic drugs. This allowed for a more focused and robust comparative analysis between the two most frequently discussed groups of antidepressants.

### Content analysis process

This study applies an unsupervised learning approach named topic modeling to detect groups of tweets inside the Spanish and English databases. After thoroughly revising the available unsupervised methodologies, Latent Dirichlet Allocation (LDA) was selected due to its widespread use, interpretability, and extensive application in X datasets within the literature [52–54].

Before applying the unsupervised algorithm, text preprocessing was essential to ensure an optimal model performance. The first step involved separating Spanish tweets from English tweets, allowing LDA to be applied independently to each dataset. Then, tweets in each dataset are refined by removing stopwords, duplicate terms, and non-standard characters such as emojis or hashtags.

Determining the number of topics in each dataset was crucial before implementing LDA. A Cluster Validity Index (CVI) was employed to identify the optimal number of topics. CVIs are metrics used in unsupervised learning to assess the effectiveness of clustering by evaluating the arrangement of data points [55]. In this research, the Silhouette Coefficient [55] was the CVI selected due to its ability to measure inter-cluster and intra-cluster distances. For each dataset, we ran LDA five times with the number of topics ranging from 2 to 10. We then calculated the mean Silhouette Coefficient for each topic number across the five iterations and selected the number of topics with the highest mean score [56, 57]. With the optimal number of topics identified, we applied LDA to both the Spanish and English datasets. Finally, using LDA, we extracted the most relevant words for each topic, which allowed us to identify the theme and assign an appropriate name to each topic based on these key terms.

In the final stage, emotion detection was conducted using a model from Hugging Face’s machine learning platform, specifically the “Emotion English DistilRoBERTa-base” model [58] for the English dataset. This model is considered state-of-the-art for detecting Ekman’s six basic emotions – anger, disgust, fear, joy, sadness, and surprise – along with the neutral emotion [59]. It has demonstrated an accuracy of 66%, significantly surpassing the 14% probability of random chance (1/7). For the Spanish tweets, we used the Robertuito model [60], which achieved an accuracy of 58%. Both models are built on the RoBERTa architecture; however, DistilRoBERTa employs a distilled version to reduce complexity, while Robertuito uses a full RoBERTa model with 12 self-attention layers, 12 attention heads, and a hidden size of 768 to replicate the structure of BERTweet. Both models were pre-trained to classify the emotions of interest, so no additional training was required.

### Ethical aspects

This study was approved by the Research Ethics Committee of the University of Alcalá and follows the ethical guidelines of the Declaration of Helsinki (2013). Since it used publicly available tweets, no human subjects or interventions were involved. However, we ensured user anonymity by not disclosing names or including tweets that could reveal identities.

## Results

### Total count of tweets

A total of 1,570,321 tweets mentioning antidepressants in both Spanish and English were collected from 2007 to 2022. Of these, 1,048,576 were in English and 521,745 in Spanish. After excluding 35,448 English and 86,199 Spanish tweets that either did not meet the inclusion criteria or were unintelligible, a final dataset of 1,448,674 tweets was analyzed: 1,013,128 in English and 435,546 in Spanish.

**Figure 1.**
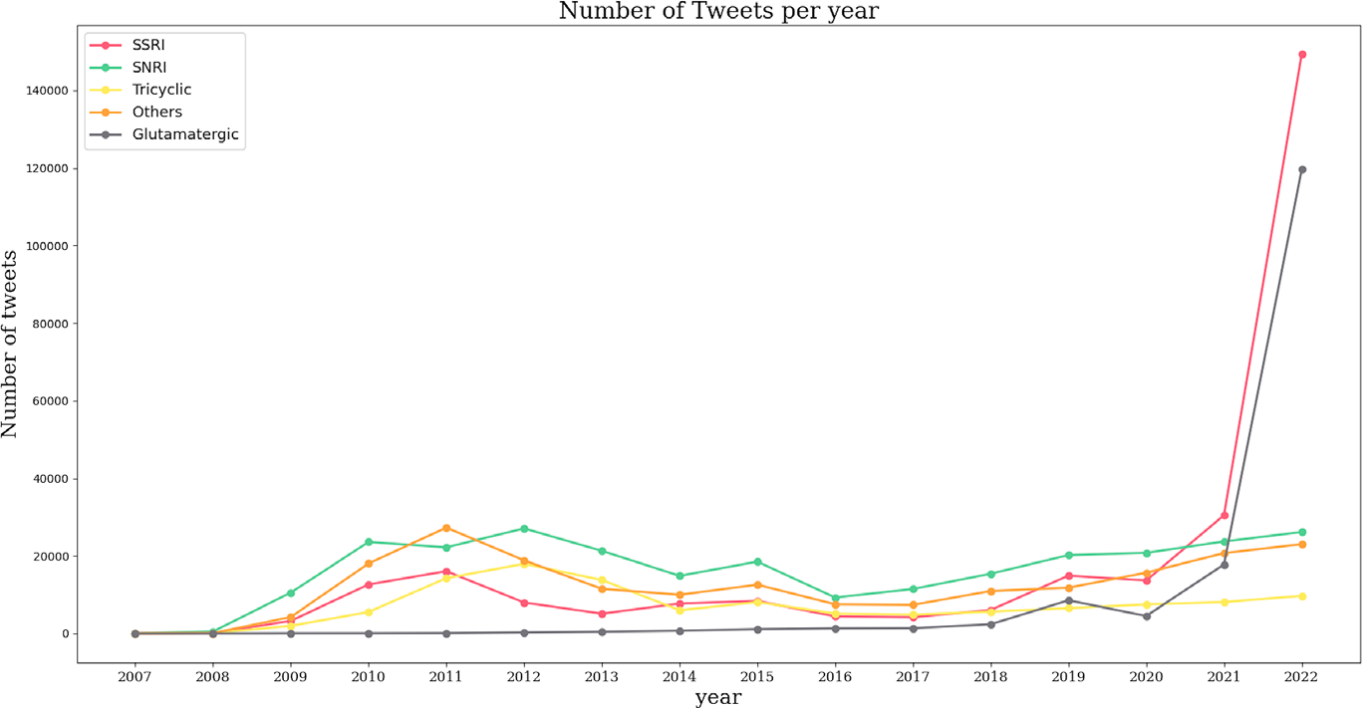
Number of tweets in English per year per drug. Each antidepressant group has its own color represented in left superior corner of the panel.

**Figure 2.**
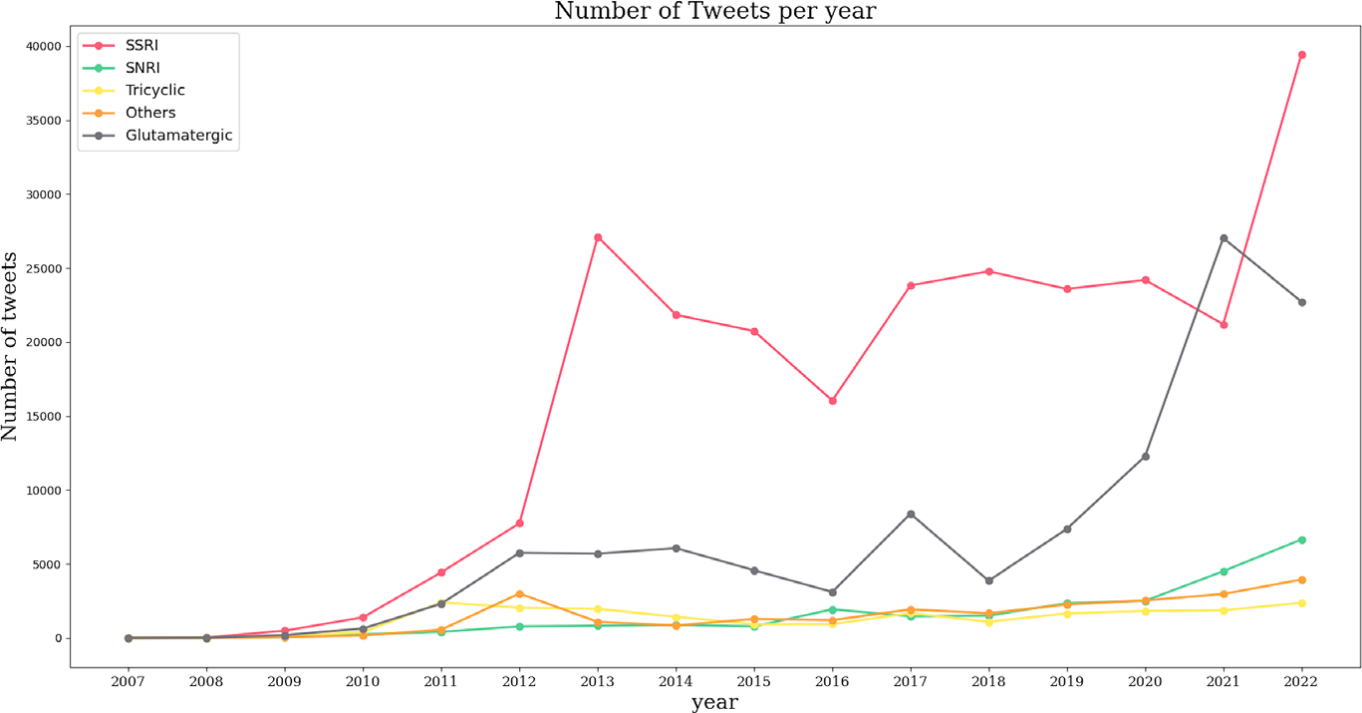
Number of tweets in Spanish per year per drug. Each antidepressant group has its own color represented in left superior corner of the panel.

SSRIs emerged as the most frequently mentioned class of antidepressants in both languages. In English tweets (Supplementary Material – Figure 1) SSRIs accounted for 27.9% of all mentions, while in Spanish tweets (Supplementary Material – Figure 2), they constituted a significantly higher proportion at 58.91%. Variations were observed in the mentions of SNRIs, TCAs, and other classes of antidepressants between the two languages. In English tweets, SNRIs represented 25.71%, TCAs 11.28%, and other antidepressants 19.5%. In contrast, Spanish tweets showed lower proportions for SNRIs (5.72%), TCAs (4.74%), and other antidepressants (5.39%). Glutamatergic antidepressants were mentioned more frequently in Spanish tweets, representing 25.24% of all mentions, compared to 15.61% in English tweets.

### Number of tweets per year

In English-language tweets, mentions of antidepressants remained stable between 2007 and 2020, followed by a significant increase in references to SSRIs from 2021 onwards (Figure 1). A similar upward trend was observed for glutamatergic drugs starting in 2021. Notably, prior to the rise in SSRI mentions, dual agents accounted for the highest volume of tweets.

In contrast, Spanish-language tweets primarily focused on SSRIs, with notable peaks in 2012 and 2021. Between 2013 and 2016, there was a marked decline in SSRI mentions, followed by a resurgence in 2021. Tweets referencing glutamatergic drugs showed a steady increase beginning in 2011, peaking in 2017, and experiencing exponential growth between 2018 and 2021 (Figure 2).

### Topic modeling analysis of SSRIs and glutamatergic antidepressants

**Figure 3.**
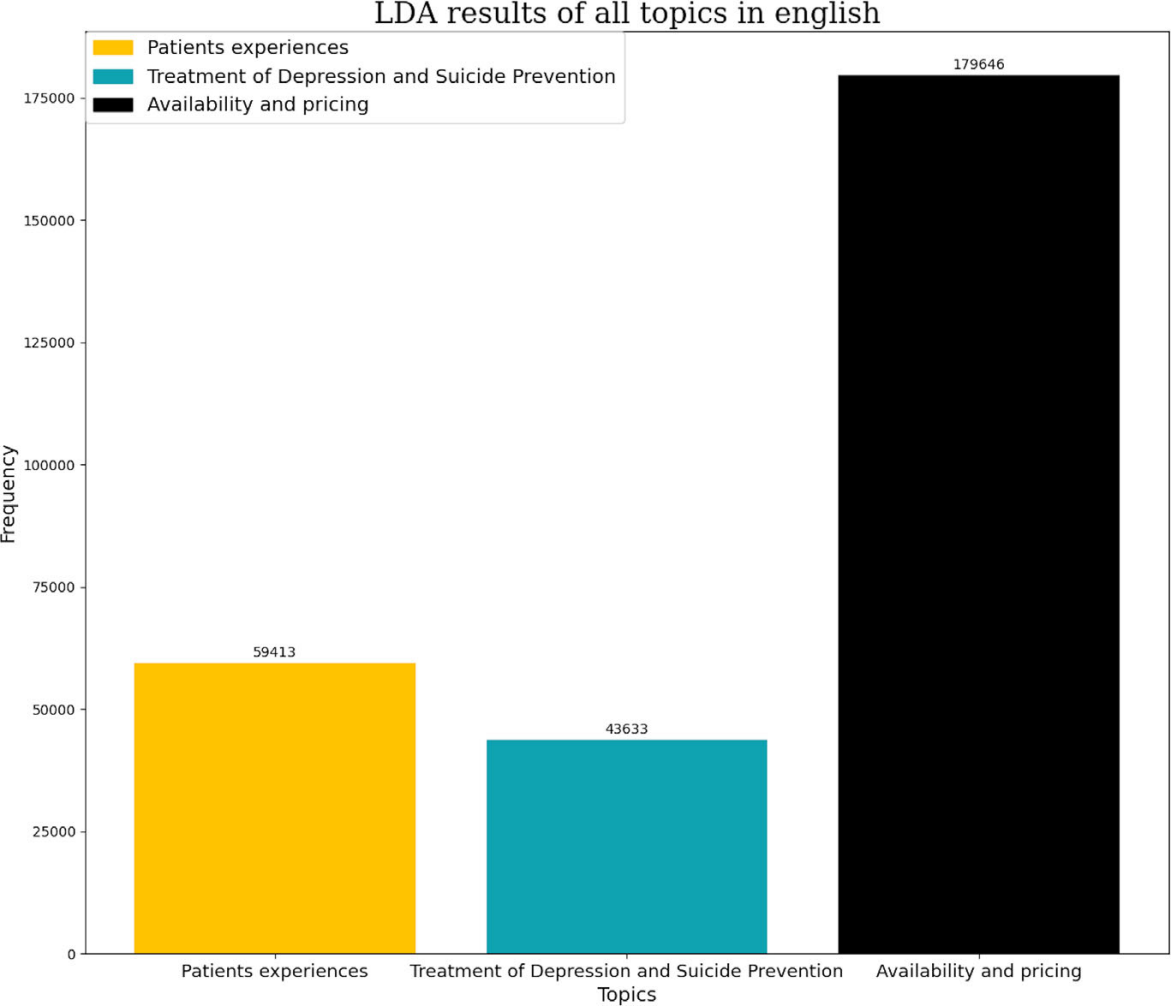
Number of tweets per topic in SSRI in English. Each topic has its own color represented in left superior corner of the panel.

**Figure 4.**
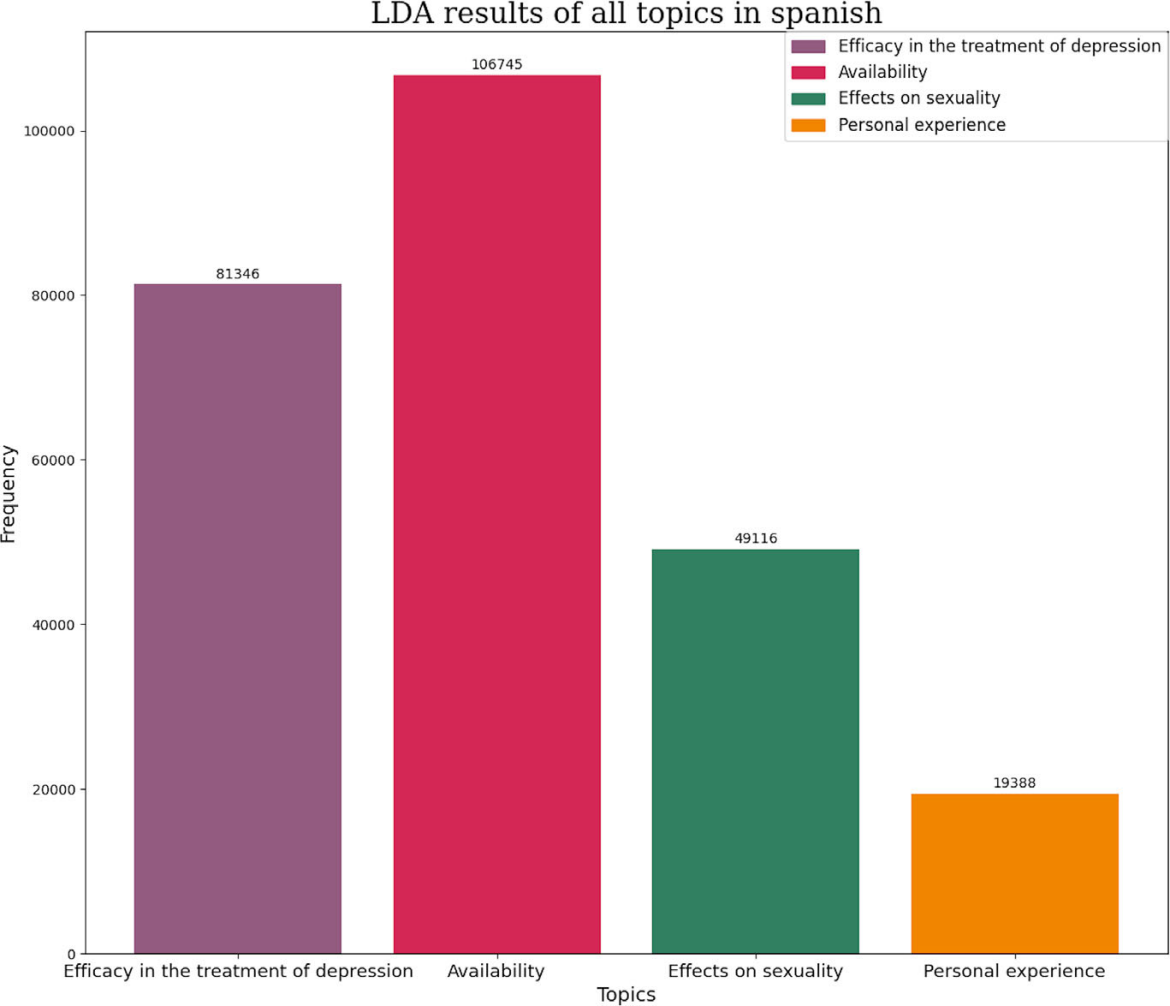
Number of tweets per topic in SSRI in Spanish. Each topic has its own color represented in left superior corner of the panel.

Following a topic modeling analysis, the most frequent themes were classified based on the number of tweets and the corresponding pharmacological group. In English, the themes related to SSRIs were availability and pricing (63.55% of tweets), personal experiences (21.02%) and lastly tweets related to depression treatment and suicide prevention (15.43%) (Figure 3). In Spanish, the themes with the highest number of tweets were availability (41.6%), efficacy in depression treatment (31.7%), effects on sexuality (19.14%), and lastly personal experiences (7.56%) (Figure 4).

**Figure 5.**
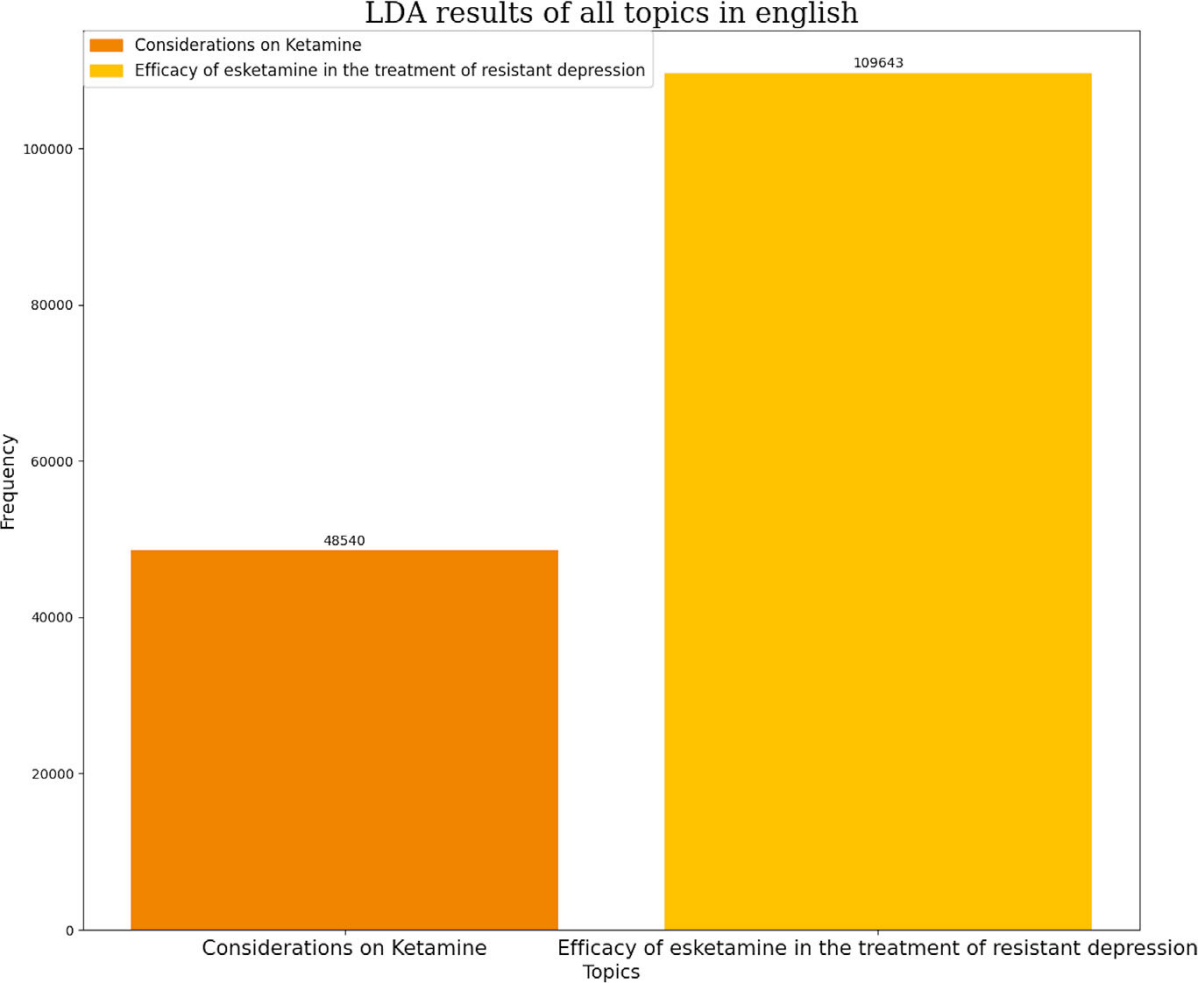
Number of tweets per topic in glutamatergic drugs in English. Each topic has its own color represented in left superior corner of the panel.

**Figure 6.**
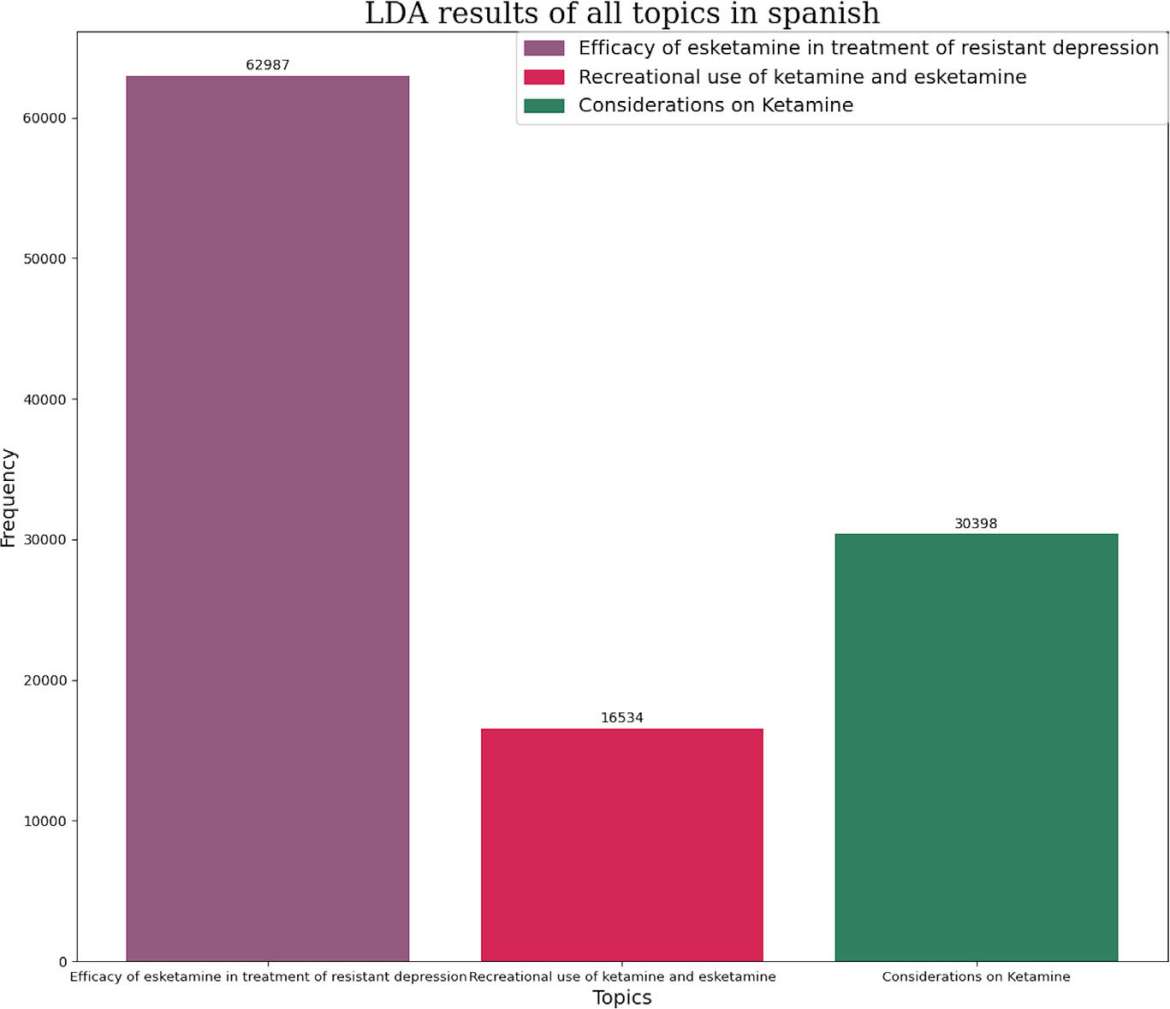
Number of tweets per topic in glutamatergic drugs in Spanish. Each topic has its own color represented in right superior corner of the panel.

Similarly, the most frequent themes in tweets discussing glutamatergic drugs were classified. In English, the most frequent themes were the efficacy of esketamine in TRD (69.31%) and considerations regarding ketamine use (30.69%) (Figure 5). In Spanish, themes included the efficacy of glutamatergic drugs in TRD treatment (57.3%), considerations regarding esketamine (27.7%), and recreational use of ketamine and esketamine (15%) (Figure 6).

### Temporal analysis of the main topics related to SSRIs and glutamatergic drugs

**Figure 7.**
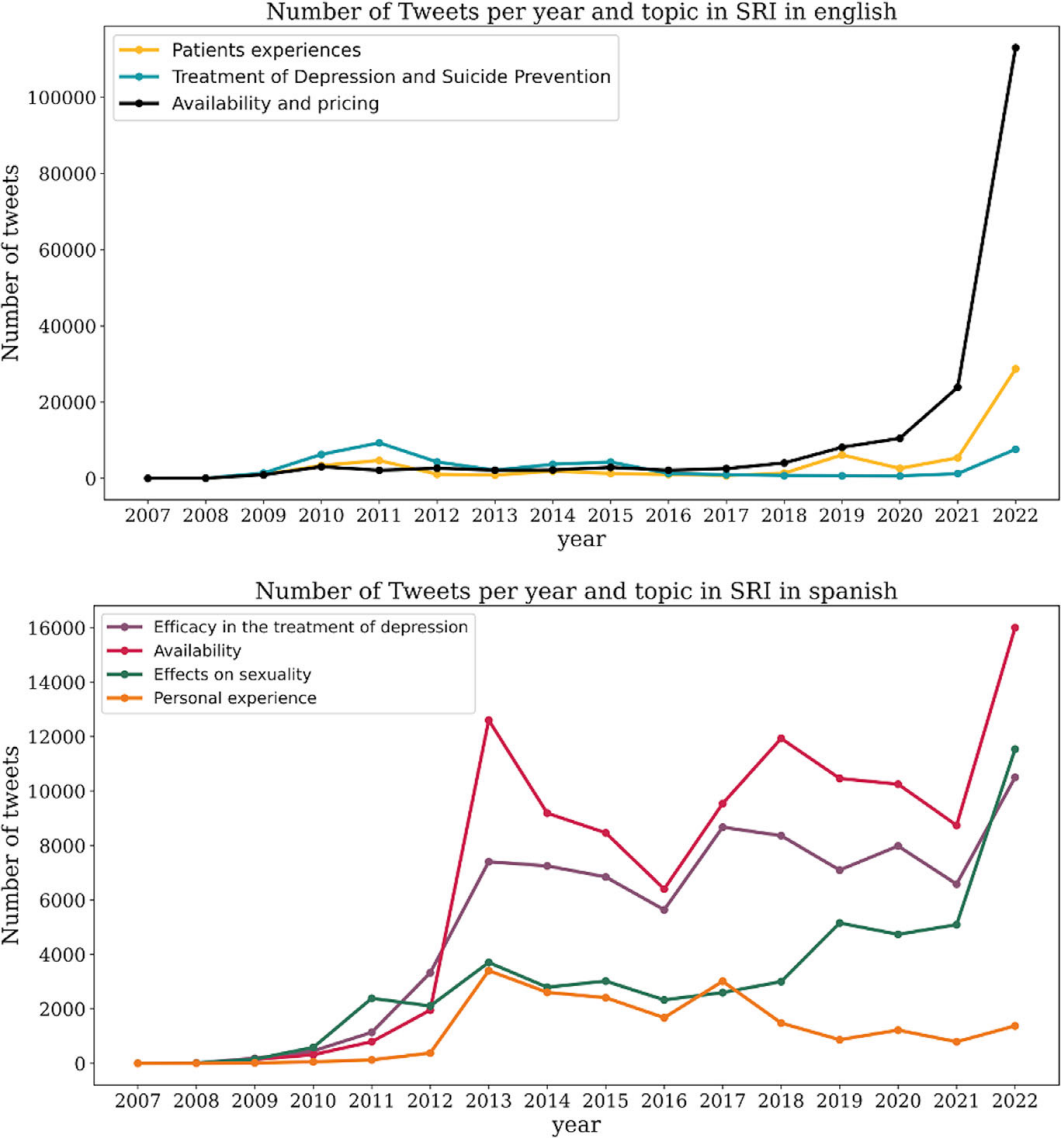
Temporal evolution of the number of tweets related to SSRIs, per year and topic, in English (superior panel) and Spanish (bottom panel).

**Figure 8.**
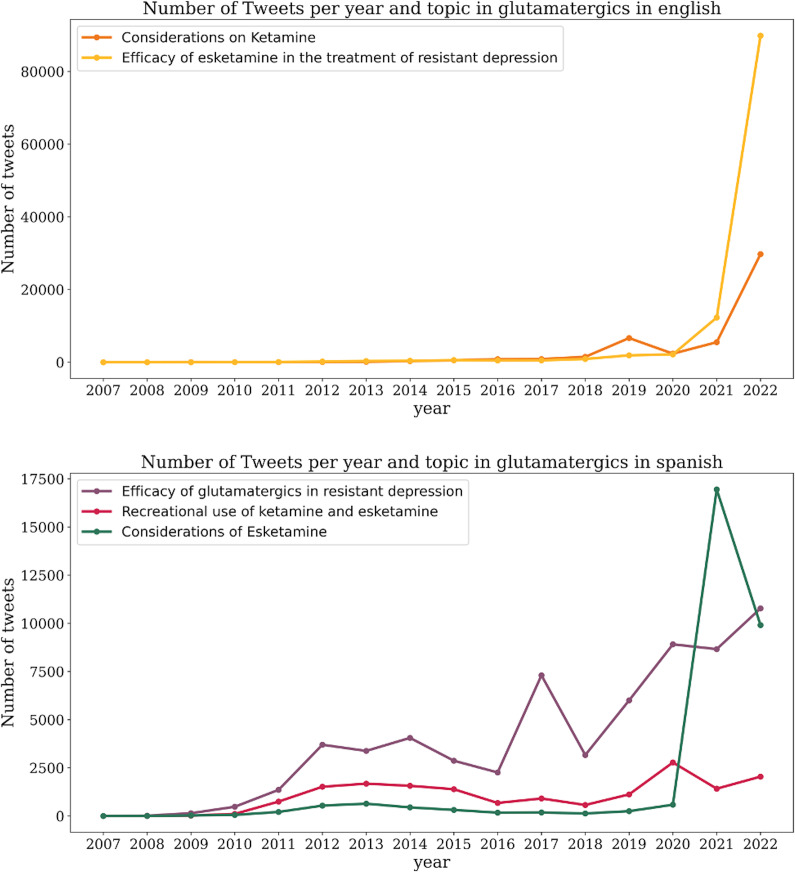
Temporal evolution of the number of tweets related to glutamatergic drugs, per year and topic, in English (superior panel) and Spanish (bottom panel).

We analyzed the evolution of different topics related to SSRIs and glutamatergic drugs in both English and Spanish tweets. In English, the three primary themes remained relatively stable over time, with a slight increase in tweets discussing depression treatment and suicide prevention noted in 2011 (Figure 7). From 2020 onwards, there was a significant rise in tweets related to availability and pricing (Figure 5a). Similarly, starting in 2021, there was a notable surge in tweets about glutamatergic drugs, particularly the efficacy of esketamine for treatment-resistant depression (Figure 8).

In contrast, Spanish tweets showed greater fluctuations and thematic diversity over the years. An increase in tweets about availability was observed in 2013, remaining steady until another peak in 2022. Discussions on the effects of SSRIs on sexuality grew gradually from 2010, with a more pronounced increase in 2022. Similarly, tweets about the efficacy of SSRIs in treating depression showed a steady rise from 2010, peaking again in 2022 (Figure 7). Tweets about the efficacy of glutamatergic drugs in treatment-resistant depression also steadily increased, with a marked rise in discussions about esketamine starting in 2021 (Figure 8).

### Sentiment analysis for each topic

**Figure 9.**
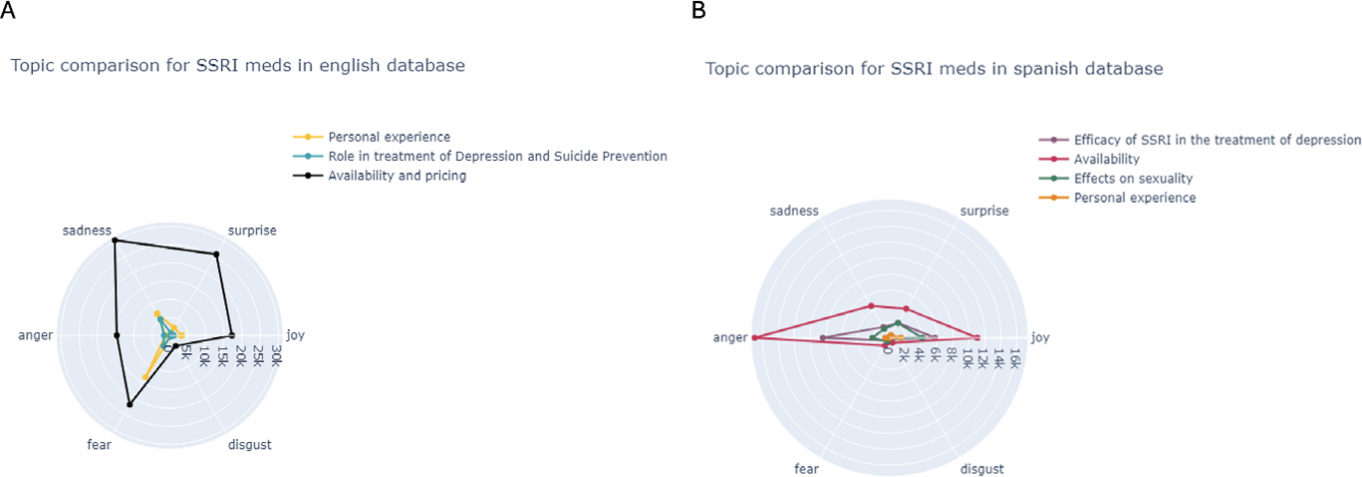
Sentiment analysis of the number of tweets per year and topic in English (A) and Spanish (B) tweets from SSRIs.

Figure 9A shows that in English tweets about SSRIs, availability and pricing elicit sadness, fear, and surprise, while personal experiences are dominated by fear, and sadness prevails in discussions of depression treatment and suicide prevention. In Spanish tweets (Figure 9B), anger dominates conversations about availability and efficacy, while joy is more prominent in discussions on sexuality and personal experiences.

**Figure 10.**
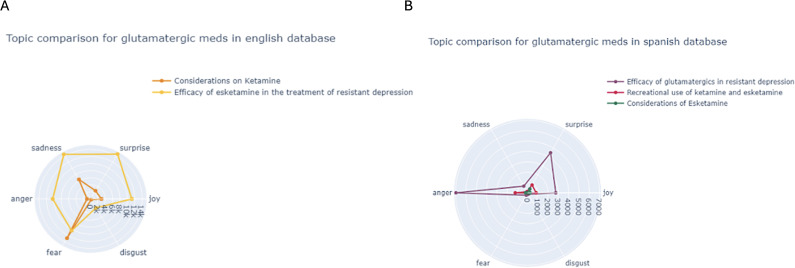
Sentiment analysis of the number of tweets per year and topic in English (A) and Spanish (B) tweets from glutamatergic drugs.

For glutamatergic treatments in English (Figure10 A), fear and sadness are the main emotions in tweets about ketamine, while esketamine’s efficacy in treatment-resistant depression evokes surprise, sadness, and some joy. In Spanish, anger is the predominant emotion in tweets about esketamine’s efficacy in treatment-resistant depression and its recreational use, followed by surprise and joy in both cases (Figure 10B).

## Discussion

In this study, we analyzed over a million English and Spanish tweets posted between 2007 and 2022 to investigate public perceptions of antidepressants, focusing on SSRIs and glutamatergic modulators. Using artificial intelligence techniques such as sentiment analysis and topic modeling, we identified key concerns, emotions, and cultural differences in the discussions surrounding these medications. Our findings reveal that SSRIs were the most frequently mentioned antidepressants in both languages, with pricing and availability being key concerns, particularly in English tweets, while Spanish tweets often emphasized efficacy and sexual side effects. Additionally, discussions around esketamine for treatment-resistant depression gained traction in recent years, with notable differences in emotional responses between languages. These findings provide real-time insights into public attitudes, highlighting the influence of social and cultural contexts on the perception and acceptance of antidepressants, especially in light of the growing global focus on mental health.

SSRIs are the most widely used antidepressants globally, recommended as first-line treatment for depressive disorders [11, 12, 61] and a range of psychiatric conditions [62]. This broad use likely explains their prominence in both English and Spanish tweets, a trend seen in previous studies [51]. Notably, in Spanish tweets, we observed two distinct peaks: one in 2013 and another, in 2022. The 2013 increase aligns with national reports from Spain, which documented a 200% rise in antidepressant use between 2000 and 2013, with SSRIs showing a 159.3% increase [63] likely tied to the economic crisis and the arise of mental health issues during this period. Both language groups showed a significant rise in SSRI-related tweets between 2020 and 2022, coinciding with the COVID-19 pandemic, which drove a global rise in SSRIs antidepressant prescriptions [64, 65], particularly among adolescents and young adults [66].

Our analysis of tweets discussing SSRIs revealed that drug availability was a key concern in both languages, with anger dominating Spanish tweets and sadness, fear, and surprise being more common in English tweets. These emotions reflect real-world challenges like shortages and price increases during the pandemic, especially in countries like the UK and the United States [67, 68]. Similar concerns have been reported in Latin American countries, where shortages of medications, particularly antidepressants, have raised alarm [69].

In English tweets, the availability and pricing of SSRIs were also major topics of discussion on X, particularly in 2021 and 2022. By 2020, the cost of prescribing SSRIs in countries like the United Kingdom had tripled compared to 2019, largely due to shortages of active pharmaceutical ingredients and the increased cost of generic drugs during the pandemic [70]. Similarly, the United States saw drug costs rise, with Medicaid expenditures on antidepressants increasing from $1 billion in 2017 to $1.12 billion in 2021, with SSRIs accounting for the largest share [71, 72]. In contrast, the lower focus on pricing in Spanish tweets might reflect differences in healthcare systems, such as the broader coverage provided by Spain’s public healthcare system. These findings highlight how X mirrors key societal issues, but further research is needed to fully understand the interplay between language, region, and healthcare context in shaping these discussions. Previous studies have proposed using social media information as a pharmacovigilance tool due to its immediacy and timeliness [68].

The efficacy and use of SSRIs were frequently discussed in both languages, but with notable emotional differences. Spanish tweets often expressed anger, along with some joy and sadness. The prominent anger may be attributed to the delay in SSRIs’ therapeutic effects, as these medications typically take several weeks to become effective [73]. This delay can lead to frustration or anger among patients seeking immediate relief.

Previous research by Leis et al. [74] found that patients undergoing SSRI treatment showed small but statistically significant increases in happiness and surprise emotions, without significant changes in sadness, anger, fear, or disgust. This contrast with the lower levels of joy observed in our study, emphasizing the nuanced emotional responses associated with SSRI treatment. Interestingly, Leis et al. also reported a slight increase in the use of negations during the treatment period, potentially reflecting ongoing concerns or dissatisfaction. Further research is needed to better understand the underlying reasons for these emotional reactions, as this knowledge could provide valuable insights for clinicians when managing patient expectations and experiences with these medications.

In contrast, English tweets focused more on the role of SSRIs in suicide prevention, a topic that remains controversial. Many English tweets expressed fear and sadness, reflecting ongoing public concern about the potential link between SSRIs and increased suicidal ideation, particularly in adolescents and young adults. Since 2004, regulatory agencies have issued warnings about this risk in younger populations [75], and although meta-analyses since 2009 have examined the relationship between antidepressants and suicidal thoughts [11], the evidence remains inconclusive [76]. While some studies suggest efficacy in treating depression but an increased risk of suicidal acts in young people, others show no increased risk in adults, with some even indicating protective effects against suicide in adults [76–81].

The emotional tone in these discussions highlights the complexity of the issue and the need for further research. Social media platforms like X could play a vital role in detecting depressive symptoms and suicidal behaviors [82, 83], particularly among vulnerable groups like adolescents, making this an important area for future research.

Many drugs, such as antidepressants, can cause adverse effects if abruptly discontinued [84], making early detection of drug shortages through social media potentially beneficial for both patients and healthcare professionals. A prominent theme in Spanish tweets, not present in English tweets, was the impact of SSRIs on sexual function, with emotions primarily reflecting joy, surprise, and anger. While the anger may be related to the well-documented sexual side effects of SSRIs, such as reduced libido and sexual dysfunction [85–88], these associations should be interpreted with caution, as they cannot be definitively established based solely on social media data. Similarly, the expressions of joy and surprise could be linked to the use of SSRIs for premature ejaculation, where they are often prescribed due to their safety, tolerability, and efficacy [89, 90].

Emotional tones in tweets are influenced by a variety of factors, including individual experiences, cultural attitudes toward discussing sexuality, and the healthcare context in different Spanish-speaking regions. The higher volume of discussions about sexual effects in Spanish tweets suggests strong public interest in this topic, highlighting the importance of providing patients with clear information about these potential side effects before initiating treatment.

Since 2019, there has been a significant increase in tweets mentioning glutamatergic drugs, especially esketamine, in both English and Spanish. This surge aligns with the FDA and EMA approval of esketamine for TRD in 2019 [91], indicating that discussions on X reflect ongoing clinical developments. Esketamine, notable for its novel mechanism targeting NMDA receptors and its intranasal administration, has gained attention as a promising option in TRD and other psychiatric conditions, [92, 93]. Our analysis shows that both English and Spanish tweets primarily focused on the efficacy of esketamine in TRD, though Spanish tweets also emphasized glutamatergic modulators in general. While short-term efficacy is well-supported by evidence [94, 95], uncertainty remains regarding long-term outcomes, with mixed research findings on sustained benefits [96–98]. Esketamine is better than placebo in the relapse rate and the remission rate at 32 weeks of follow up [96] and although it is approved in the majority of European countries, it has not been approved in the United Kingdom by the Nice guidelines [98]. These doubts also extend to ketamine, influencing public perceptions and emotional responses on social media.

A recent study by Ng et al. [99] found a shift in public attitudes after esketamine’s approval, with discussions centered on regulatory changes, cautious optimism, and positive personal experiences, which partially align with our findings. Sentiment analysis revealed that, in English tweets, emotions like surprise and sadness dominated, though joy was also present, reflecting hope and optimism regarding esketamine and ketamine treatments. In contrast, Spanish tweets showed more anger, likely due to skepticism or frustration over high costs and limited access, but joy was also evident, indicating that these treatments still inspire hope among Spanish-speaking users.

Interestingly, we found that recreational use of ketamine and esketamine was the third most commented topic in Spanish tweets, but not in English ones. Recreational ketamine use has risen in recent years, with a significant increase in ketamine seizures in the United States between 2017 and 2022 [100]. Spain has also seen a rise in use, with 0.9% of the population reporting ketamine use at least once between 2020 and 2022 [101]. South America has reported emerging trends of ketamine use, including its combination with other substances, such as in the new concoction “Tusi” (composed of ketamine and often combined with substances like MDMA, methamphetamine, cocaine, opioids, and new psychoactive substances), which has gained popularity in multiple regions [102].

The increase in recreational use is reflected in social media discussions, where our sentiment analysis found anger to be the predominant emotion, followed by surprise and joy. Anger may stem from concerns about the trivialisation of ketamine’s therapeutic uses, as has occurred with other medications that have also become recreational drugs [103, 104]. However, a 2022 study by Grabski et al. [105] found no significant changes in public perceptions of ketamine’s risks following the approval of esketamine for medical use. They highlight the importance of clearly communicating the risks of recreational ketamine use in discussions about its therapeutic benefits. Further research into these recreational uses on social media platforms like X is warranted.

This study relied solely on X data, which may not represent the broader patient population, as X users tend to be younger and more tech-savvy. To strengthen the analysis, other social media platforms or traditional research should be considered. Tweets’ character limits can lead to incomplete or unclear messages, often missing key context about the user’s condition or treatment. Variations in drug names and misspellings can also distort sentiment. Additionally, since only Spanish and English tweets were analyzed, the findings may not apply globally.

Cultural attitudes influence the emotional responses seen in this study, highlighting the need to consider context when evaluating public perceptions of psychiatric treatments as antidepressants. Differences between regions sharing the same language may further shape these perceptions, reflecting variations in healthcare systems, social norms, and access to treatments.

Our results emphasize the importance of further research and spreading accurate information on social media to improve perceptions and reduce misunderstandings. On X, antidepressants, especially SSRIs, are discussed with optimism and concerns about availability, pricing, personal experiences, and efficacy, shaped by cultural differences. Glutamatergic modulators, like ketamine and esketamine, have drawn attention for TRD, with Spanish tweets noting concerns about recreational use. This study shows social media’s potential to complement traditional research, though its limitations call for cautious interpretation and more research to improve its role in pharmacovigilance and public health.

## Supporting information

Chart-Pascual et al. supplementary material Chart-Pascual et al. supplementary material

## Data Availability

The datasets generated and analyzed during the current study are available from the corresponding author upon reasonable request. Additional details regarding the data collection and variables analyzed can be found in the supplementary material provided with this manuscript.

## Supplementary material

The supplementary material for this article can be found at http://doi.org/10.1192/j.eurpsy.2025.10.
