# Supplementary material for: Understanding social media discourse on antidepressants: unsupervised and sentiment analysis using X
Source: Eur Psychiatry. 2025 Mar 5;68(1):e51. doi: 10.1192/j.eurpsy.2025.10 (PMC12041734; doi:10.1192/j.eurpsy.2025.10)
Supplement: Chart-Pascual et al. supplementary material [file S0924933825000100sup001.docx]

**SUPLEMENTARY MATERIAL**

**Search strategy**

The following keywords were used in the search strategy, comprising both generic drug names and brand names: Fluoxetine, Adofen, Prozac, Luramon, Altisben, Aremis, Besitran, Ferbrain, Semonic, Citalopram, Citalvir, Prisdal, Seropram, Seregra, Escitalopram, Cipralex, Esertia, Diprex, Elicea, Heipram, Paroxetine, Arapaxel, Daparox, Frosinor, Motivan, Seroxat, Fluvoxamine, Dumirox, Vilazodone, Viibryd, Venlafaxine, Arafaxina, Dislaven, Dobupal, Levest, Vandral, Venlabrain, Venlamylan, Venlapine, Zarelis, Desvenlafaxine, Pristiq, Enzude, Duloxetine, Cymbalta, Dulotex, Oxitril, Uxagam, Dulvas, Xeristar, Mirtazapine, Rexer, Afloyan, Trazodone, Deprax, Bupropion, Elontril, Zyntabac, Ketamine/Esketamine, Ketolar, Spravato, Vortioxetine, Brintellix, Imipramine, Tofranil, Amitriptyline, Deprelio, Clomipramine, Anafranil.

**Figure 1**


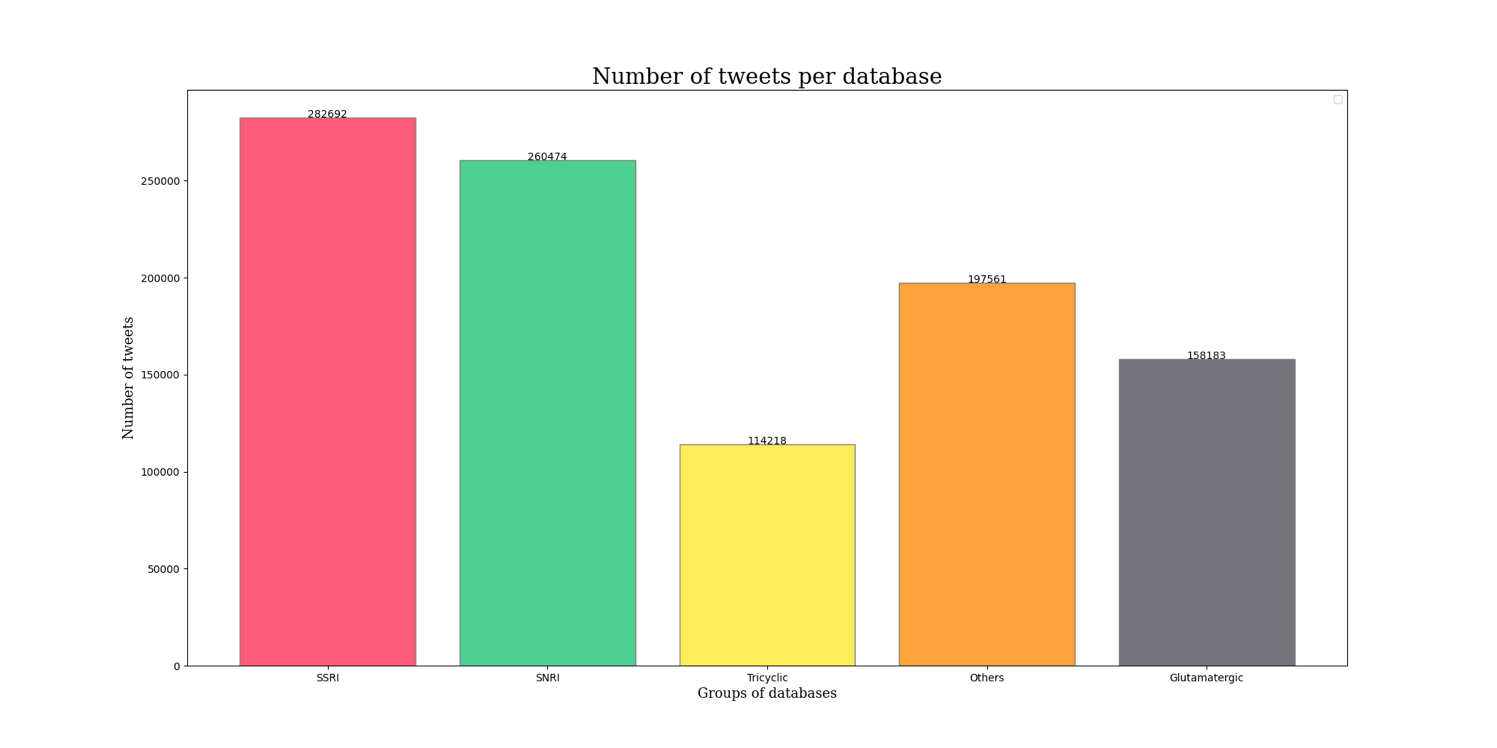


Legend: Number of tweets in English per drug Published between January 1, 2007, and December 31, 2022.

**Figure 2**


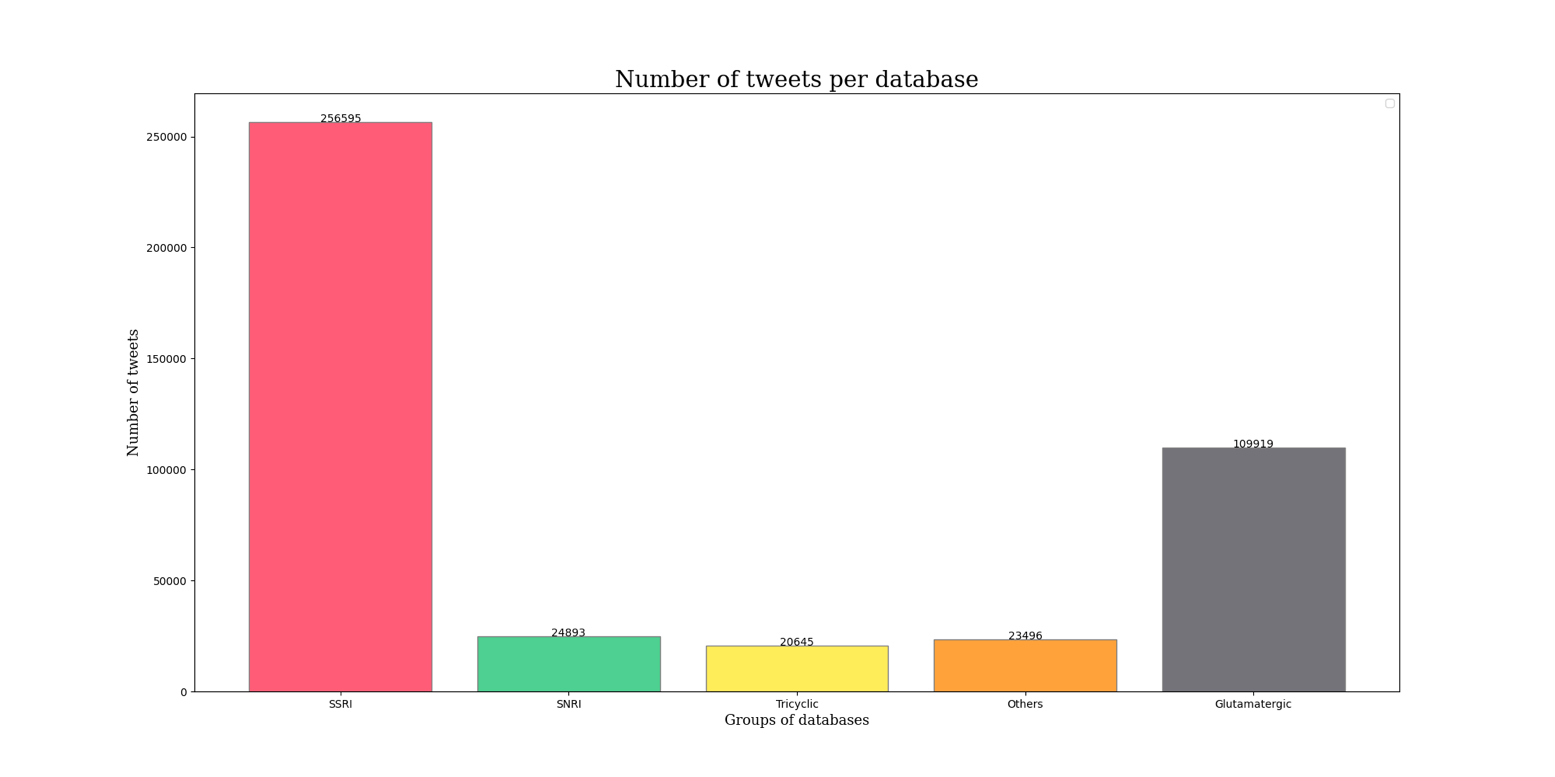
Legend: Number of tweets in Spanish per drug Published between January 1, 2007, and December 31, 2022.
